# The acceptability and feasibility of peer worker support role in community based HCV treatment for injecting drug users

**DOI:** 10.1186/1477-7517-5-8

**Published:** 2008-02-25

**Authors:** Josephine Norman, Nick M Walsh, Janette Mugavin, Mark A Stoové, Jenny Kelsall, Kirk Austin, Nick Lintzeris

**Affiliations:** 1 Turning Point Alcohol and Drug Centre, 54 Gertrude St, Fitzroy, Victoria, Australia; 2 Centre for Epidemiology and Population Health Research, Macfarlane Burnet Institute for Medical Research and Public Health, 85 Commercial Rd, Melbourne, Victoria, Australia; 3 Victorian Drug User Organisation (VIVAIDS), 128 Peel St, North Melbourne, Victoria, Australia; 4 Drug Health Services, Sydney South West Area Health Service Page Building 5, Missenden Road, Camperdown, NSW, Australia; 5 Department of Epidemiology and Preventive Medicine, Monash University Alfred Campus, 89 Commercial Rd, Melbourne, Victoria 3004, Australia

## Abstract

Hepatitis C is the most common blood borne virus in Australia affecting over 200 000 people. Effective treatment for hepatitis C has only become accessible in Australia since the late 1990's, although active injecting drug use (IDU) remained an exclusion criteria for government-funded treatment until 2001. Treatment uptake has been slow, particularly among injecting drug users, the largest affected group. We developed a peer-based integrated model of hepatitis C care at a community drug and alcohol clinic. Clients interested and eligible for hepatitis C treatment had their substance use, mental health and other psychosocial comorbidities co-managed onsite at the clinic prior to and during treatment. In a qualitative preliminary evaluation of the project, nine current patients of the clinic were interviewed, as was the clinic peer worker. A high level of patient acceptability of the peer-based model and an endorsement the integrated model of care was found. This paper describes the acceptability of a peer-based integrated model of hepatitis C care by the clients using the service.

## Introduction

Hepatitis C (HCV) affects over 200 000 Australians [1]. IDU is the most common mode of transmission of HCV in Australia – accounting for approximately 80% of all HCV cases [2], and over 90% of newly acquired HCV infections [3]. The prevalence of HCV in the injecting drug user population in Australia has been consistently estimated at between 50 and 70% since the 1970's [4-8]

Antiviral therapy for chronic HCV infection has improved considerably in recent years, with sustained virological response – equating to a probable cure – increasing from 15–20% with interferon monotherapy to 50–80% with pegylated interferon and ribavirin therapy [9]. Despite these advances in HCV antiviral treatment, the number of people accessing therapy remains small – in excess of 2000 people undergo treatment per year, representing only 1% of Australians with chronic HCV infection [1], Anecdotal evidence suggests that limited numbers of injecting drug users receive HCV treatment in Australia, even though current IDU was removed as an exclusion criterion for government-funded HCV antiviral treatment in Australia in May 2001.

There is international recognition of the need for a more inclusive approach to HCV treatment to increase the availability of treatment for patients who have previously been ineligible or difficult to engage in treatment [10]. Indeed, increasing international evidence suggests that HCV positive substance dependent injecting drug users can achieve favourable antiviral treatment outcomes when engaged in comprehensive substance use treatment and supportive HCV treatment programs [11-15].

Recently there has been an effort to reduce the barriers to HCV treatment in Australia (e.g., removing IDU as an exclusion criterion for treatment, removal of the requirement for a liver biopsy prior to commencing treatment). Despite this, limited numbers of injecting drug users have received treatment in Australia and IDU has been identified as a major barrier to receiving referrals to specialist HCV clinics or subsequently receiving HCV treatment [16]. Other Australian studies have shown that knowledge of HCV treatment options among injecting drug users is low and that medical support for HCV is often perceived as a low priority in the lives of injecting drug users [17].

HCV treatment in Australia is funded through a federal government program known as the s100 Highly Specialised Drugs Program. In 2003 provisions were made available for the delivery of HCV treatment under shared care arrangements between suitably trained community-based medical practitioners working in collaboration with a specialist physician. With the aim of moving some HCV treatment services away from hospital-based clinical settings, this development enabled HCV treatment to be situated in alternative settings such as drug treatment clinics where large numbers of injecting drug users could potentially better access services.

The Healthy Liver Clinic (HLC) was established in 2006 as part of the Turning Point Alcohol and Drug Centre's clinical services. Turning Point is a multidisciplinary drug and alcohol outpatient treatment service in Fitzroy, an inner city neighbourhood of Melbourne. Turning Point provides a range of drug treatment services, including pharmacotherapy services (methadone, Suboxone), medicated detoxification, counselling, psychological treatments including cognitive behavioural therapy, forensic and outreach services. A key part of the HLC model was the concurrent availability of anti-viral treatment for HCV and opioid substitution therapy (methadone, buprenorphine) for opioid dependence. The rationale for pairing such services was to improve the awareness and attractiveness of these services to people with substance use problems, and to better engage and retain such patients in treatment.

The HLC team consists of sessional medical practitioners, a visiting specialist physician, peer worker, nurse and pharmacist, with access to other Turning Point allied health professionals as required. The HLC provides the following services: education and support; clean injecting equipment and risk reduction information; assessment of hepatitis serology and liver function; immunisation for hepatitis A and B; anti-viral treatment for HCV; and treatment of comorbid substance use and mental health conditions. In order for patients to access HCV treatment under the Highly Specialised Drugs Program (thereby reducing costs to dispensing fees only), patients must be HCV RNA positive, not be pregnant, be aged 18 years and over, and have no history of interferon treatment. Patients undergo a comprehensive assessment of their substance use, medical, psychiatric and psychosocial conditions prior to commencing HCV treatment, and have regular clinical reviews. There is a weekly after-hours support group facilitated by HLC staff. A peer education officer employed by a local state-based drug user organisation provides a fundamental role in the ongoing support of HLC clients prior, during and following their treatment schedule. The engagement of a peer worker in the HLC aimed to facilitate referrals and recruitment to the service, provide support to people considering and undergoing treatment and enhance patient adherence and support within the service.

Treatment is in accordance with current guidelines: 24 weeks pegylated interferon ribaviron combination therapy for genotypes 2 and 3 and 48 weeks combination therapy for other genotypes (the vast majority being genotype 1). Key virologic outcomes are achievement of an undetectable viral load at end of treatment (ETR) and 24 weeks post-treatment (SVR).

There is scant literature of participants' perspectives with regards to this kind of HCV treatment model. In addition, a key aspect of the HLC service not included in other like-models of care was the inclusion of a peer worker, employed by an external drug user organisation, operating as part of the HLC team. The aim of this evaluation was to examine both service user and peer worker perspectives regarding the integrated substance use/HCV treatment service model, with a particular emphasis on the role of the peer worker in this service. In addition, the evaluation aims to provide addiction and HCV specialists with information to facilitate improved health care relationships when treating patients with substance use problems for HCV.

## Methods

The study used qualitative methods of data collection and analysis. Semi-structured interviews were conducted with service users (group interviews) and with the HLC peer worker by an independent evaluator external to the operations of the HLC. The evaluation was approved by the Human Research Ethics Committee at the Victorian Department of Human Services.

### Client interviews

A convenience sample of nine HLC clients was interviewed about their decision to come to the HLC for HCV treatment, their impressions of the HLC and the role of the peer worker, including advantages and disadvantages of such a role. Data was collected during two group interviews lasting approximately one hour each. Five clients undergoing HCV treatment participated in the first group interview, and four clients who were eligible and waiting to commence HCV treatment participated in the second group interview. Clients were reimbursed AUD$30 cash to compensate for their time. At the time of the interviews (Sept 2006), 11 clients had commenced HCV treatment at the HLC, and 17 were eligible (completed screening) and due to commence treatment.

### Peer worker interview

The HLC peer worker was interviewed about the development and current components of their role and future directions for the service. Participating in the evaluation as a method for informing future improvements to the HLC model was an explicit part of the peer worker's role. The peer worker is also a contributing author on this paper.

### Analysis

The client and peer worker interviews were digitally recorded and transcribed. These were initially analysed for themes and issues using Nvivo 7™. The evaluators presented findings from the qualitative interviews to a panel of experts including key members of the HLC team and experts in HCV, addiction medicine and evaluation. This discussion informed the conclusions of this report and located the findings within the local and international context.

## Results

### Perceptions of the HLC and the role of the Peer Worker

Prior to engaging with the HLC few participating patients had sought treatment. Key reasons for not seeking treatment included negative experiences when enquiring about HCV treatment, lack of 'natural' alternatives to medical treatment and the stage of their condition. Most clients said they would not have started treatment without the HLC, whereas others identified that they would have attended hospital clinics if their conditions deteriorated "*as a last resort"*. One person was unaware of publicly funded HCV treatment and thought her only option was to somehow find the money for private treatment, which she estimated at around $20,000.

Clients engaged in treatment and those eligible for treatment had a positive perception of the HLC. Clients spoke about having confidence in the treatment and treatment team and feeling at ease and comfortable at the HLC. For example, one client reported "*I don't worry when I'm here"*. A number of clients stated choosing the liver clinic for treatment because it was not a hospital. In general clients felt there was no stigma attached to attending the HLC and considered staff to be non-judgmental, knowledgeable, friendly and dependable. Continuity of care in terms of seeing the same treating doctors and nurses and building relationships was viewed as a key advantage of HCL. Having the HLC embedded within a specialist alcohol and drug treatment centre was also viewed as beneficial. Comments included *"making it easier"*, not having to *"run around"*, and having multiple needs met at the one place; *"...my needs are met in a whole lot of different ways, from personal to support, to my addiction to ramifications from the addiction. And um, there is a GP here as well...you can be treated for other stuff." *The regular facilitated support groups were considered a crucial aspect of the HLC, especially if kept small and among people at approximately the same stage of treatment.

When discussing the role of the HLC peer worker, clients' narratives centred more on the broader supportive aspect of this role than on the actual tasks performed by the worker (e.g., providing information, driving clients to appointments, attending appointments with clients as requested, reimbursing pharmacy fees, arranging supplementary therapies). For many of the clients, having access to a peer support worker made the screening and treatment processes easier. Indeed, clients felt that it was essential for the program, stating: *"...I think she has made the difference between sticking to this or not...", "...I have had times where I have thought fuck this I'm not doing this anymore, go shove it up your arse, but if she wasn't here there would have been many more times...", "...if we didn't have the peer support worker this program wouldn't be running..." *The broad supportive role which clients described for the peer worker may reflect the complex needs of substance using clients in which notions of support that go well beyond a biomedical framework to include social and psychological issues such as acute situational crisis and a variety of substance use issues.

Key components of the role (as perceived by clients) were the peer worker's capacity to empathise with clients and humanise treatment. *"I reckon she puts a human face on this" *and *"She's not a doctor or anything, but a normal person... like you and me"*. Other attributes were mentioned, including the worker's ability to listen and provide good advice, her kindness, and non-judgmental attitude. A few clients said that having this role helped clients and doctors communicate, as illustrated by the following exchange:

Client A: "Well the doctors are in touch basically by having the peer worker there. She or he can tell the doctor exactly what the patients are feeling and get through to them."

Client B: "Yeah. And for some people it might be easier to talk to someone like [the peer support worker] rather then the doctors so you still get the information across."

Client C: "And some people feel threatened by the doctors and they can talk to [the peer support worker] and let the doctors know how they feel."

The peer worker described her role as *"helping to identify barriers to treatment, then identify and provide the support required... the rationale is to remove barriers to treatment, we have the 'too-hard' group." *She reported that much of her support included listening and lengthy deliberations to help clients weigh up the pros and cons to *"plot their own position on treatment"*. The peer worker spoke of providing information and advice to existing and potential clients and other substance use agencies about a broad range of clinical and logistical considerations relating to HCV treatment. She talked about the importance of offering practical support, often in the form of transporting clients to appointments and acted as an advocate within Turning Point in establishing a no-cost treatment scheme when clients alerted her to the financial burden of ancillary drugs.

The peer worker saw most clients face to face about once a week and attended clinical consultations at the HLC or elsewhere if requested. For clients with limited transport options or who were heavily impacted by the medication, the peer worker spoke of offering to drive them to and from home and appointments. The peer worker said her contact with clients was predominantly over the telephone and included being 'on-call' after hours. She said she received two or three after-hours calls a week, which sometimes involved her going out to meet clients in person.

### Perceived benefits of a peer worker

Perceived benefits of incorporating a peer worker role in the HLC model were explored. The fact that the peer worker had similar life experiences was important for some clients. One client contrasted their interactions with the peer support worker as opposed to a member of the medical team highlighting that nothing needed to be hidden from a peer:

"I think the fact that she has been there makes you feel that you don't have to hide anything from her. She is not judging you. She won't will she? Everything she gave us or however we carried on with our habits is something that she does know. Like [the peer support worker] will sort of say, 'I can't tell you not to use but I will say that if you are going to use do it safe, cut your dose down or something like that. The things [medical team member] said 'in an ideal world if this was my chance I wouldn't touch anything'. We'll see that coming from a person who doesn't really understand addiction, even though he smokes cigarettes."

A different view was put forward by one person, who felt that the benefit came from the personal qualities of the peer worker instead of her life experiences. She explained *"I think it is about who she is as a person, a lot of it, it not just that she has been through things."*

To tease out how important the shared life experience was to the clients, both groups were asked to imagine they were writing a position description for the HLC Peer Support Worker, and asked should peer experience be an 'essential' or 'desirable' element. The majority considered it desirable (instead of essential), although all said that peer experience was important.

The peer worker highlighted the importance of the role by stating *"...it's an integral part of treatment because the drugs are so savage. You can't just send clients away to deal with it alone because it just sets them up to fail. Even for clients who don't strike me as needy, it's a terrifying process and without [the support role], they'll get through on good luck not good practice."*

The peer worker was asked the same question as the clients – could a non-peer worker do this role? Her answer was unequivocally *"No"*. In justifying this response, the peer worker stated that one of the most important aspects of the role was that clients could discuss openly any drug use:

"Strengths of peer-based services is that there is no us and them, that I identify with the clients which is essentially different to even a very supportive non-peer worker.... We've ended up attracting lots of ex-users which makes it more of a minefield, lots of them haven't completed recovery and there are major issues about lapses and relapses. There are all these things about being open, wanting to be abstinent which means they feel that lapses can't be part of the process... A client who makes a bid to stop using and lapses, feels like a failure, they worry that they've let [medical staff] down. All of that makes it very difficult for them to be honest... Whether the judging is real or perceived, it's still very real to clients and it's a huge divide to engage across. Having a peer worker in the role means that I know this territory because it's my reality too. A lot of what I talk to clients about is drug use. I'm very pragmatic, shit happens, people lapse. Lots of clients on treatment have profound fears that drug use will impact on treatment and they can't talk to clinicians about this, I clarify that this isn't going to undermine the treatment, it's about being stable."

### Are there problems with the peer worker model?

We were interested in seeing if clients felt there were any problems in having a peer worker in this support role. The issue of the peer worker's drug use arose. The most consistent message was that current drug use by the peer worker was not a problem for clients, although some clients added that if they had a peer worker who was currently using, they would want to know about this use. Furthermore, a client differentiated between stable and unstable use rather than use per se.

In one group of clients currently in treatment, the issue of trusting a peer worker was discussed in some detail between a few clients. One client felt that drug users were inherently untrustworthy, a sentiment with which another two strongly disagreed. One client had concerns that *"poor behaviour" *by a peer worker would not have the same professional ramifications for a peer worker as for another person. Alternately, another client observed that she expected the peer worker to be both professional and a peer.

The peer worker identified a number of concerns with this clinic model and the support role of a peer worker. She noted that for *"very needy" *clients, it might *"encourage or indulge a dependency dynamic"*. For example, one client had requested the peer worker to provide all of their transportation to and from clinic appointments. Another identified problem of being (or having) a peer worker in the role was the potential distrust some clinicians in the broader medical and health sector have of peer workers: *"they [medical professionals] don't welcome peers as professionals ... there are constant complaints about me nodding off."*

### How does the peer worker role fit in with the rest of the Healthy Liver Clinic team?

The positioning of the peer worker role within the rest of the HLC team produced some interesting points of discussion. Most clients felt that the role was squarely embedded within the treating team, but interestingly, a few clients (with more experience of HCV treatment), said that the peer worker was *"right beside me" *and could be both inside and outside the team depending on the situation. One client discussed the importance of this flexibility that enabled the peer worker to advocate to the medical staff on behalf of the client. The same client said that the peer worker was also seen as the main person they spoke with about drug use or cravings.

The peer worker identified her relationship with other HLC staff as follows:

"Within the Healthy Liver Clinic team I was pleasantly surprised. I have a different perspective from the doctor's but I am given a voice. My fear that my role would be tokenistic hasn't been my experience at all and the clinicians do consult with me."

The peer worker did, however, identify the need for a professional framework for the support role: *"Sometimes I feel like I'm making it up as I go along and the stakes are high, sometimes I feel out of my depth"*. Clients identified the complexity of the peer worker role in this service model (of being both a member of the HLC team and a confidante for the clients), and the need for independent professional clinical supervision. The peer worker reported that this was particularly difficult in relation to discussions of drug use with clients *"There were times when I've felt very conflicted about how and what I tell the others [rest of the Healthy Liver Clinic team]...I mean, at what point is drug use a problem."*

The peer worker also identified the importance of being employed and linked to a user group organisation with experience in peer worker models, highlighting the support she received through such an arrangement. *"...given broader organisational issues with peer workers, I would be lonely and isolated if I wasn't part of VIVAIDS [the Victorian drug users community organisation], ... need people to run things by..."*. Managing the logistics of having two workplaces was, however, somewhat problematic, but was identified as *"a small price to pay."*

### Future directions

In order to help clients articulate what they did and did not value in the HLC model, we asked them how they would set up a second HLC. Clients said the following elements were important and should be included in any new HLC: an on-site pharmacy, blood tests, psychologist, doctors and one peer worker per 10 clients. Also, where possible, peer workers should be employed in a variety of roles such as reception and administration. The offer of transportation was also mentioned and some clients thought this could be provided by the peer worker or a junior staff member chosen by the peer worker. Clients felt it was important for the clinic to have a humanistic, genuine, honest, non-judgmental and calm environment that considered the emotional well being of patients. Other suggestions included hiring committed and specialist staff and continue to build relationships *"one-on-one" *between clients and staff. Regarding future developments, clients identified the need for expansion of the service by increasing the number of staff in the HLC and/or setting up similar clinics elsewhere.

The peer worker identified that, although the initial model for the HLC meant that it could be *"truly responsive,"* it was also somewhat naïve, labour intensive and needed to be revisited in light of the demand for services. The peer worker identified the need for a more structured approach regarding the amount of support provided to clients: *"In order to provide this level of support, we need to work out what that involves. We need to give clients a reasonable idea of the level of support they can receive. We should be saying at the beginning that 'I might see you once a week for an hour' and if they think they'll need more support, we might need to tell some of them that we can't support them through this."* She identified that in order to accommodate an increased demand for HLC services by clients with considerable support needs, the HLC should increase the capacity of staff (i.e., increasing the size of the team, hours available) and/or enable clients to provide a higher level of support to one another. Given appropriate support and training clients could facilitate the support group and receive 'train the trainer' expertise to utilise their knowledge and experience. The peer worker identified a model whereby one peer worker be employed for every 25 clients in HCV treatment.

Recognising the contextual importance for a treatment model such as this, the peer worker identified the need to incorporate HCV issues into all assessments of injecting drug users in specialist or GP settings, promote access to ongoing monitoring (screening every 6–12 months), and recognise that treatment is not the only valid option. In relation to government policy, she felt there is a *"...huge onus on providing treatment for hepatitis C, however no money to deal with any increased demand for treatment. All the tertiary clinics are at capacity and the process is so slow that bloods [blood tests] are having to be duplicated. If that's the status quo, what happens if injecting drug users start wanting treatment?"*

## Discussion

The available literature provides increasing evidence that injecting drug users can achieve equivalent outcomes from HCV treatment compared with non-users [18-27]. Because it takes between 12 and 18 months to complete HCV treatment and find out if sustained virological response has been achieved, we are unable to provide data on treatment effectiveness at this stage. However, treatment compliance rates have been excellent (10 of 11 in treatment with 100% attendance records), and we have no reason to believe that the HLC will not deliver treatment outcomes comparable to other studies of injecting drug users.

The HLC model provides peer support through an employed peer worker and a support group. Although peer-based drug treatment and harm reduction programs are wide spread, particularly those focusing on BBV prevention, there is a scant amount of literature assessing feasibility and acceptability of peer based hepatitis C treatment models. Despite this, the HLC model is not unique in the literature [13,28]. Although these studies have described the use of peers in hepatitis C treatment programs as useful in attracting patients to treatment and providing counselling, as Madden describes in a recent editorial [29] there are systemic barriers in many countries to peer based organisations gaining funding to contribute to mainstream services.

This evaluation demonstrates the acceptability of this model in a number of key areas. Clients were satisfied with the knowledge and support provided by the peer counsellor during their engagement with the clinic, regardless of whether they were on HCV treatment or not. It is clear that the individual characteristics of the peer worker, beyond simply being a peer, played a key role. It is not just about getting 'any' peer worker. For many (but not all) clients, however, it was the 'peer' experience that provided sufficient validation to enable increased understanding and empathy in addition to the rest of the team. Although the benefits of a peer worker role in the team were clearly identified, issues such as confidentiality and supervision were important themes that arose and need further examination. For example, to what extent should a peer worker have to divulge their own drug use to clients (as identified in some client interviews) or other health professionals? The peer worker spoke of the distrust she sometimes perceived from health care workers. Somewhat ironically, this distrust and the general stigma experienced by IDU within healthcare settings provided a significant rationale for the establishment of the HLC and the role of the peer worker in a community-based liver clinic. Also, at what point should a peer worker divulge client information (such as a client's substance use) to other members in the team, especially when the impact or consequences of the substance use may not be readily apparent to the client or peer worker (such as the impact upon hepatic or immune function)? In this context, the need for ongoing and independent clinical supervision for the peer worker is clearly important.

A client centred treatment model for a population of individuals with complex needs should optimally involve an integrated treatment approach. The HLC model requires an interdisciplinary team with high levels of staff expertise and integration. The HLC model incorporates substance use, psychiatric and psychological treatment, infectious disease and/or gastroenterology support, primary health care and an informed onsite pharmacy team within a harm reduction context. The model attempts to mirror the patients' needs, rather than the patient having to fit the treatment model which is so often the case. In this regard it is also important to recognise that clients attending a liver clinic may do so to primarily evaluate their HCV rather than to receive treatment. This point might be particularly relevant for people choosing to attend a community based clinic and for IDUs whose current life circumstances may recommend a delay in commencing treatment and who may have had limited opportunities to engage clinicians and receive information about their HCV [16,17]. This point was touched on by the HLC peer worker who endorsed an ongoing management approach to HCV care for IDU in other specialist and GP settings.

Many of the principles underpinning the establishment of the HLC model (e.g., mutual respect, including patients in decision making, multidisciplinary team providing continuity of care), have been endorsed by others when discussing the engagement of drug users in health care relationships [10,30]. The complex needs of active users, or those with a recent history of IDU that embark on HCV treatment require medical, psychiatric and substance use support and treatment to address the varied clinical issues that arise. Whereas biomedical problems are often the primary concern among patients with less complex needs, problems encountered by this population include broader medical, social and psychological issues such as acute situational crisis, depression, hypomania, anaemia, neutropenia, and a variety of substance use issues. As such, sufficient and multidisciplinary clinical supervision and adequate initial competency are prerequisites for a successful program.

It is apparent that this service model is also resource intense. Although it can be argued that HCV treatment of injecting drug users will have cost benefits to the community and health services over time [31], this does not necessarily translate to funding for the services. Funding schemes for HCV treatment such as the one that operates in Australia often cover essential medical and medication costs, but do not fund the broader range of support services identified as being important by this group of clients, such as a peer worker. Thus, existing funding mechanisms may be adequate to accommodate individuals with less complex treatment needs but are unlikely to meet the needs of injecting drug users that make up the largest category of patients in need of HCV treatment. Much of this problem stems from the intersectoral divide between the various components of health that HCV bridges – infectious disease, substance use, crisis support and mental health. Integrated service models require integrated funding.

In addition to feasibility issues of this model with regards to recurrent funding, the sustainability of what was identified as an extremely labour-intense peer worker role needs to be considered. While endorsing the "responsive" nature of the care model, the peer worker recommended that future models should explicitly define the nature of the support to be provided and make clear to clients the limitations of such support. Such considerations need to ensure the sustainability of a model of care and should pragmatically take account of financial constraints discussed above, who pays for and employs the peer worker, the potential for peer worker "burn-out", the expected number of clients and the complexity of their needs, the training needs of support staff and the number of support staff that could realistically be employed.

There are limitations to this study that are worth noting. It is a small convenience sample of clients – although it does represent almost half of the clients who had undergone HCV treatment at the time of the interview. This group of clients had only ever experienced HCV screening and treatment under this model – and it may be that alternative models (e.g., without a peer worker, but a professional welfare worker) may have been as favourably received. As a result there is sample bias in the statement that clients preferred the community based liver clinic to hospital treatment for this reason. In addition, whereas clients received this service positively, the clinical effectiveness of the HCV treatment provided will need to be assessed in order to validate the model as clinically appropriate. Finally the peer worker is a named author in this paper. Given the intimate involvement of the peer worker in the clinic, the evaluation was designed in conjunction with the peer worker and thus these efforts should be recognised. The peer worker was not involved in the analysis to reduce conflicting interests.

## Conclusion

In conclusion, this evaluation has demonstrated that the peer-based integrated model of HCV care for injecting drug users is acceptable and feasible to the clients who are engaged in the service. The information presented here is a valuable addition to the literature of HCV treatment provision to injecting drug users that can be used or adapted by other clinical programs to best design services to this patient group. There is an increasing literature on integrated care models, particularly in the context of HIV, but so far little on HCV. Despite the complexity of the patient profile, this evaluation demonstrates that it is certainly feasible to provide a quality, integrated HCV treatment service that is acceptable to substance using clients.

## Competing interests

Jenny Kelsall would like to acknowledge a potential conflict of interest in being a co-author on this paper and being the peer worker who was evaluated in this paper. Jenny Kelsall and all authors would like to emphasise that the information presented in this paper is a true representation of participant responses. Jenny Kelsall did not contribute to the analysis to minimise this conflict of interest. The authors have no other competing interests to declare.

## Authors' contributions

JN conceived study design carried out, analysed the research and coordinated the writing of the paper, NW contributed to study design, provided supervision, technical advice and was involved in writing the paper, JM contributed to study design, data collection and writing of the paper, MS contributed to study design, data analysis and writing of the paper, JK provided technical input in study design. KA provided support to the research team, study participants and advice on writing the paper, NL provided technical advice, supervision and was involved in writing the paper. All authors read and approved the final manuscript.
